# Analysis of working and health conditions in street vendors of Bogotá, Colombia, in the context of the COVID-19 pandemic

**DOI:** 10.47626/1679-4435-2023-1203

**Published:** 2024-11-14

**Authors:** Jaime Moreno-Chaparro, Olga Beatriz Guzmán-Suárez, Mónica Bermúdez-Lugo, Johan Sebastian Muñoz-González, Laura Alejandra Sánchez-Avellaneda

**Affiliations:** 1 Occupation and Social Inclusion Research Group, Faculty of Medicine, Universidad Nacional de Colombia (UNAL), Bogotá, Colombia; 2 Human Occupation Department, Faculty of Medicine, Universidad Nacional de Colombia (UNAL), Bogotá, Colombia

**Keywords:** social determinants of health, COVID-19, informal sector, Colombia, employment, determinantes sociales de la salud, COVID-19, sector informal, Colombia, empleo

## Abstract

**Introduction:**

The COVID-19 pandemic caused an economic, social and health crisis that, despite the lifting of restrictions in the so-called “new normality,” resulted in increased vulnerability and informal employment.

**Objectives:**

To analyze the working and health conditions of a group of informal workers who develop their economic activities in the streets of Bogotá in the context of the COVID-19 pandemic and of the so-called post-pandemic new normality.

**Methods:**

A mixed methods study was conducted on a sample of street vendors by applying a standard questionnaire and a qualitative phenomenological analysis. Data were analyzed descriptively, sub analysis was performed according to economic activity, and significant associations were tested.

**Results:**

A total of 191 street vendors of low socioeconomic status were included. Of note was the predominance of workers affiliated to the subsidized social security system (p = 0.012) and the fact that more than 89% were not affiliated to other protection systems. Participants perceived that their income decreased after the pandemic (50-80%) and that the wage-work relationship was unfair (p = 0.045). The health-work relationship was explored in categories such as challenges during the pandemic, work concerns, and work well-being.

**Conclusions:**

The working, employment and health conditions of street vendors in the context of the pandemic worsened their already precarious living conditions and work flexibility, exposing them to adverse situations such as an increased risk of severe acute respiratory syndrome coronavirus 2 infection and a constant need to earn a living wage.

## Abstract (es)

**Introducción:**

La pandemia de COVID-19 provocó una crisis económica, social y sanitaria que, a pesar del levantamiento de las restricciones en la llamada “nueva normalidad”, se tradujo en un aumento de la vulnerabilidad y del empleo informal.

**Objetivos:**

Analizar las condiciones laborales y de salud de un grupo de trabajadores informales que desarrollan sus actividades económicas en las calles de Bogotá en el contexto de la pandemia de COVID-19 y la llamada nueva normalidad postpandemia.

**Métodos:**

Se realizó un estudio de métodos mixtos sobre una muestra de vendedores ambulantes en el que se aplicó un cuestionario estándar y un análisis fenomenológico cualitativo. Los datos se analizaron de forma descriptiva; se realizaron subanálisis según la actividad económica, y se probaron asociaciones significativas.

**Resultados:**

Se incluyó un total de 191 vendedores ambulantes de nivel socioeconómico bajo. Se destacó el predominio de trabajadores afiliados al régimen subsidiado de seguridad social (p = 0,012) y el hecho de que más del 89% no estaba afiliado a otros sistemas de protección. Los participantes percibieron que sus ingresos disminuyeron tras la pandemia (50-80%) y que la relación salario-trabajo era injusta (p = 0,045). La relación salud-trabajo se exploró en categorías como desafíos durante la pandemia, preocupaciones laborales y bienestar laboral.

**Conclusiones:**

Las condiciones de trabajo, empleo y salud de los vendedores ambulantes en el contexto de la pandemia empeoraron sus ya precarias condiciones de vida y flexibilidad laboral, exponiéndolos a situaciones adversas como un mayor riesgo de infección por síndrome respiratorio agudo severo 2 y una necesidad constante de ganar un salario digno.

## INTRODUCTION

Latin America and the Caribbean (LAC) is a geopolitical region where countries share some social, cultural, economic and health conditions that, in the context of the COVID-19 pandemic, led to a higher frequency of unfavorable situations for most of their inhabitants and to an increase of both inequality and inequity in the region. The above is evidenced in indicators such as: a contraction of the economic activity of at least -7% by 2020 and 2021, the closure of at least two million micro and small enterprises, the loss of approximately 26 million jobs, the increase of multidimensional poverty indices, and the poor and fragmented response of health and social protection systems in the context of the health and economic crisis caused by the pandemic, which has made LAC one of the most critical regions in the world.^1,2^

After COVID-19 was declared a pandemic in March 2020, several biosafety (e.g., frequent handwashing, permanent use of facemasks, among others) and social distancing (human mobility restrictions, lockdowns, suspension of social gatherings, closing of schools and the mandatory implementation of distance learning, etc.) measures were implemented by most governments around the world to slow down the spread of the disease.^3^ However, despite the fact that more than 2 years have passed since then and that most of these measures are no longer in force, they have caused a series of damages, mainly social and economic, affecting mainly decent work and healthy work environments, access to health services, and the provision of health care to vulnerable population groups.^4^

Thus, informal employment has emerged as a response to these difficulties in LAC, as it has become the main source of income for most people in the region during the post-pandemic period (i.e., after the full reactivation of all economic and social activities), since currently up to 70% of new jobs in the region are found in the informal sector.^2^ Informal work is understood as any unregulated labor activity in which the worker has no protection or social assistance and where there is no legal protection.^5^ Informal workers are constantly exposed to a high risk of economic vulnerability and poor working and health conditions, which ultimately endangers their potential development as individuals in all aspects.^6^

Undoubtedly, the COVID-19 pandemic has increasingly exacerbated the already precarious conditions of informal workers, as they have been exposed to periods of economic inactivity and, as a result of it, to limited access to health care services (even in the primary care setting), higher rates of food insecurity (and, therefore, a higher risk of malnutrition), higher poverty rates, as well as to a higher risk of severe acute respiratory syndrome coronavirus 2 (SARS-CoV-2) infection, and therefore of post-COVID-19 sequelae (even death), compared to workers in the formal economy, given their working conditions.^4,7^

In Colombia, a South American country of at least 50 million inhabitants, there are more than five million informal workers, and informal economy is considered the main economic sector of the country; in addition, it has been described that the number of informal workers increased by approximately 268,000 between September and November 2021, and that said increase was related to the pandemic.^8^ In Bogotá, informal workers were exposed to even more precarious labor and health conditions, since, given its size and population density, government authorities implemented strict human mobility restrictions in the city that heavily affected informal workers, as many of the economic activities they were engaged were not considered to be essential and many were left without a source of income.^9,10^

Currently, most of the restrictions implemented to control the COVID-19 pandemic have been lifted, and the risk of severe illness from SARS-CoV-2 infection is low in the general population. However, so far there are no data on the transition from the implementation of the human mobility (both social and economic) restriction measures to the “new normality” after the pandemic (defined as the progressive return of society to its regular activities amid the existing threat of the pandemic) in informal workers of Bogotá explaining the current labor, health and welfare conditions in this population, as well as their interrelation with each other. Based on the above, the aim of this study was to analyze working and health conditions of a group of informal workers that carry out their economic activities in the streets of Bogotá in the context of the COVID-19 pandemic and the so-called post-pandemic new normality.

## METHODS

### ETHICAL CONSIDERATIONS

The present study was approved by the Ethics Committee of the Faculty of Medicine of the Universidad Nacional de Colombia under code 009-067 issued on May 13, 2021. All participants signed the respective informed consent form and data were anonymized, ensuring confidentiality at all times.

### DESIGN

Mixed methods study in which a standardized questionnaire was administered, and an interpretative phenomenological qualitative analysis based on semi-structured interviews was carried out.

### DATA SOURCE AND STUDY PARTICIPANTS

**Figure 1:**
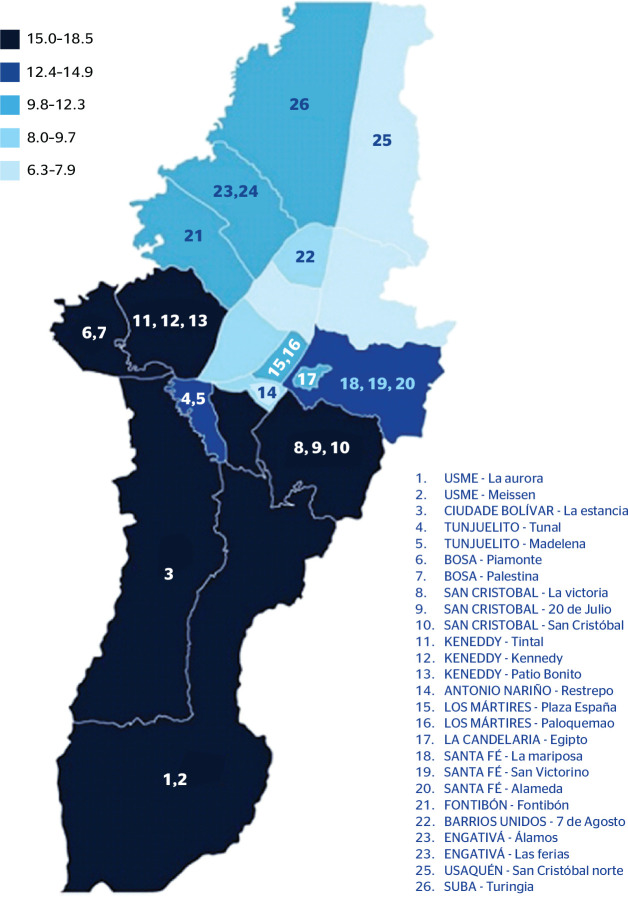
Geographic density (% of people) and location density (number) of informal workers in Bogotá. This figure was made using the data available in the 2021 Multipurpose Survey conducted by the Departamento Administrativo Nacional de Estadística (DANE).

Informal workers selling goods in public spaces of Bogotá (street vendors) were recruited between November and December 2021. The study decided to use a non-probabilistic sampling method based on geographic density and participation quotas because previously the research team found that there are specific geographic areas and localities within Bogotá with higher densities of street vendors and products or goods of importance to the local economy. Priority was given to the following localities: Santa Fe, San Cristóbal, and Kennedy (Figure 1). Furthermore, due to the wide range of street vending subcategories, depending on the goods they sell, only street vendors selling food, miscellaneous items (electronic items, hardware items, stationery items, among others), and clothes were considered.

Participants were selected according to the following criteria: 1) having worked for at least 5 years as a street vendor selling the goods informed in the identification process (seniority); 2) having worked for at least 5 years in the location where the informal worker was identified; 3) being older than 18 years; 4) being officially registered in the Institute for Social Economy of Bogotá (IPES) as a street vendor in the Informal Street Vendor Individual Registration Form; and 5) having a score < 20 points in the System for the Identification of Potential Beneficiaries of Social Protection Programs (in Spanish, Sistema de Identificación de Potenciales Beneficiarios de Programas Sociales [SISBEN]), a Colombian system developed by the Departamento Administrativo Nacional de Estadística (DANE) to measure people’s socioeconomic vulnerability.^11^

### MEASURES

Participants were administered the Basic Questionnaire on Working, Employment and Health Conditions in Latin America and the Caribbean (in Spanish, Condiciones de Trabajo, Empleo y Salud en América Latina y el Caribe [CTESLAC]). The main objective of this instrument is to monitor workers’ health taking into account basic sociodemographic characteristics and work-related and health-related conditions, as well as the presence of workplace safety and prevention resources and actions.^12^

Participants were also administered the Occupational History Questionnaire (OHQ). This instrument was designed to be used in the context of a qualitative interview based on a phenomenological-interpretative perspective that aims to collect information to build concepts and occupational narratives that make work-related situations and realities visible by the recognition, analysis and understanding of the population and its context.

### VARIABLES

#### Sociodemographic information

The following sociodemographic data were obtained: sex, age, schooling level, place of origin (only the five most frequent departments of birth are presented in the results section), current type of residence (urban or rural), socioeconomic level (it should be noted that in Colombia there are six socioeconomic strata and they are used to determine people’s level of income, as well as their access to public services, being stratum one and two equivalent to low socioeconomic level, that is, the most vulnerable individuals or those with the least access to such services), whether the worker was a victim of forced displacement, the number of years working as an informal street vendor, and the type of goods sold (food, miscellaneous items and clothes).

#### Employment and working conditions

Twenty-one questions from this component were considered for the data analysis to have an overall understanding of participants’ employment and working conditions. These questions were related to the characteristics of the participants’ work activity, compliance with the regulations of the Colombian labor system, and occupational hazards.

#### Health conditions

Data on health conditions were collected by questions about participants’ general health condition, questions of the General Health Questionnaire (GHQ-12) on self-perception of life and well-being,^13^ the World Health Organization-Five Well-Being Index (WHO-5) (mental health or psychological well-being),^14^ and questions about the occurrence of work-related injuries and occupational diseases.

#### Work-health relationship

This relationship was explored through the OHQ, specifically three questions on the impact the COVID-19 pandemic had on the participants, namely: 1) What challenges have you faced as an informal worker due to the COVID-19 pandemic?; 2) What are your concerns regarding your work as a street vendor in the context of the pandemic?; and 3) What strategies have you used to improve your working conditions and reduce the risk of infection?

#### Data collection process

The above questionnaires were administered in their Spanish versions (question and direct response *in situ*) with the possible support of a researcher previously trained (in case of difficulty of comprehension or lack of clarity). Subsequently, the qualitative interview was conducted with the constructed questions, for which the participant’s voice was recorded and then the research team carried out verbatim transcriptions. Both the questionnaire and the qualitative interview were applied to all participants; however, for the qualitative analysis, data saturation was considered.

#### Statistical methods and analysis

Sociodemographic information and the data collected by the administration of the CTESLAC questionnaire were analyzed using descriptive statistics: absolute frequencies and proportions were calculated for the description of categorical variables, and measures of central tendency and of dispersion for quantitative variables. Subsequently, sub-analyses were performed according to the street vending categories established. The chi-square test was used to determine significant associations for categorical variables, while an analysis of variance (ANOVA) was performed to establish differences between groups. A significance level of p < 0.05 was considered. All statistical analyses were performed using the R software (version 3.5.0).^15^ Qualitative analyses were carried out using the NVivo 12 software^16^ starting with the creation of delimited categories for first-level coding purposes and then with the interpretation of data and the identification of similarity patterns.

## RESULTS

A total of 191 informal workers were included. Average age was 43 years, 52.4% were women and 40.3% were natives from Bogotá. Regarding their schooling level, 85.2% reported having completed any level of education, with 37.1% having completed secondary education or high school and 35.6% having completed only primary education. Furthermore, most participants (78.5%) had a low socioeconomic status (socioeconomic stratum 1: 27.7% and socioeconomic stratum 2: 50.8%) and 10.5% were victims of forced displacement due to violence and the internal conflict. The average number of years working in the informal sector was 16.5, and there was a similar distribution of workers in the three street vending categories that were considered (food [n = 67], miscellaneous items [n = 57], and clothing [n = 67]) (Table 1).

**Table 1:** Participants’ sociodemographic characteristics and general information (n = 191)

Sociodemographic variable	Total n (%)
Sex	
Female	100 (52.4)
Age (years), mean (SD)	44.5 (13.6)
Birth department	
Bogotá	77 (40.3)
Cundinamarca	32 (16.8)
Boyacá	10 (5.2)
Caldas	10 (5.2)
Valle del Cauca	8 (4.2)
Education level	
No education	28 (14.6)
Completed elementary school	68 (35.6)
Secondary or high school completed (baccalaureate)	71 (37.1)
Technician complete	8 (4.2)
Technologist complete	12 (6.2)
Professional complete	4 (2.1)
Residence	
Urban	187 (97.9)
Socioeconomic stratum	
One	53 (27.7)
Two	97 (50.8)
Three	41 (21.5)
Displacement	
Yes	20 (10.5)
Years in the informal sector, mean (SD)	16.5 (11.3)
Main activity	
Food	67 (35.1)
Miscellaneous items	57 (29.8)
Clothing	67 (35.1)

SD = standard deviation.

### CTESLAC QUESTIONNAIRE

Significant differences were found between groups in the average number of people served in a workday (p = 0.000), the average working hours per week (p = 0.015) and the working days per week (p = 0.025). In detail, the average number of people served in a workday was higher in the group of street vendors selling food (55.8 people) and those selling clothes worked, in average, more hours per week (66.9 hours); in addition, most street vendors selling clothes worked from Monday to Friday (85.1%).

Regarding the enrollment in social security and health systems, significant associations between street vending categories (food, miscellaneous items, clothing) and the enrollment of informal workers in the subsidized social security and health system (i.e., in which health services are provided by the Colombian state for free) were found (p = 0.012). Furthermore, between 89.5 to 95.5% of all street vendors in the three groups were not enrolled in a mandatory pension fund, a workers’ compensation insurance company, and/or in a social complementary services system.

Regarding their income, more than half of the participants in each group stated that before the COVID-19 pandemic their monthly income ranged between one and two minimum wages (232-465 USD, 1 USD = 0.000256 COP) and that said income had been reduced to less than one minimum wage in the post-pandemic period, that is, the new normality (USD < $232 USD = 0.000256 COP).

Finally, risks due to exposure to solar light or solar radiation were significantly associated in all groups (p = 0.028), as was the perception of unfairness regarding the wage-work performance relationship (p = 0.045).

In relation to participants’ health condition, high scores were predominant in the general health status item (0 to 100 points), with mean average scores of 79.9, 78.1 and 78.7 points in the food, miscellaneous items, and clothing groups, respectively.

Other finding that stands out is that a considerable number of participants reported they had experienced more than usual sleep deprivation in the last month due to concerns related to their work activity. However, in contrast, most of them stated that the feelings of being under pressure (p = 0.033) or not being able to overcome difficulties had not increased in the last month in terms of frequency (p = 0.519). Findings in other variables of interest are presented in Table 2.

**Table 2:** Participants’ health, employment, and working conditions according to the results of the administration of the Basic Questionnaire on Working, Employment and Health Conditions in Latin America and the Caribbean (CTESLAC) questionnaire

	Food (n = 67) n (%)	Miscellaneous items (n = 57) n (%)	Clothing (n = 67) n (%)	p-value
**Employment and working conditions**				
How many people on average do you serve in a workday? Mean (SD)	55.8 (52.0)	28.9 (20.5)	35.4 (27.2)	**0.000**
How many hours do you work on average per week? Mean (SD)	65.4 (16.9)	58.6 (17.0)	66.9 (15.8)	**0.015**
What days of the week do you usually work?				
Monday to Sunday	46 (68.7)	40 (70.2)	57 (85.1)	**0.025**
What is your usual work schedule?				
Morning and afternoon shift	56 (83.6)	47 (82.5)	58 (86.6)	0.576
Are you affiliated to the social security health system?				
Yes, by subsidized scheme	46 (68.7)	28 (49.1)	47 (70.1)	**0.012**
Yes, by contributory scheme	14 (20.9)	17 (29.8)	14 (20.9)	
Are you affiliated to the Pension/Labor/Supplementary Social Services System?				
None of the above	63 (94.0)	51 (89.5)	64 (95.5)	0.485
Can you take holidays without any problem?				
No	48 (71.6)	36 (63.2)	57 (85.1)	**0.019**
When you are sick, do you take medical incapacity time?				
No	37 (55.2)	27 (47.4)	40 (59.7)	0.677
Do you go to the doctor when you need to?				
Yes	46 (68.7)	34 (59.6)	43 (64.2)	0.275
What was your income before the pandemic?				
< 1 minimum salary	30 (44.8)	28 (49.1)	26 (38.8)	0.608
1-2 minimum salary	36 (53.7)	28 (49.1)	41 (61.2)	
2-3 minimum salary	1 (1.5)	1 (1.8)	0 (0.0)	
What has been your income during the last few months in pandemic/post-pandemic?				
< 1 minimum salary	54 (80.6)	46 (80.7)	47 (70.1)	0.57
1-2 minimum salary	12 (17.9)	10 (17.5)	19 (28.4)	
2-3 minimum salary	1 (1.5)	1 (1.8)	1 (1.5)	
In your job, how often do you work exposed to a noise level that forces you to raise your voice?				
Always	40 (59.7)	33 (57.9)	39 (58.2)	0.218
How often do you work in your job exposed to sunlight (radiation)?				
Always	48 (71.6)	48 (84.2)	56 (83.6)	**0.028**
How often do you work with harmful/toxic chemicals in your job?				
Never	27 (40.3)	28 (49.1)	29 (43.3)	0.175
How often do you hold awkward positions at work?				
Always	24 (35.8)	22 (38.6)	22 (32.8)	0.656
Often	13 (19.4)	13 (22.8)	15 (22.4)	
In your job how often do you lift, move or drag loads, people, animals or other objects?				
Always	34 (50.7)	22 (38.6)	22 (32.8)	0.198
In your main job how often is your salary fair in relation to your job performance?				
Never	33 (49.3)	20 (35.1)	19 (28.4)	**0.045**
To what extent are you concerned about how difficult it would be to find another job, should you become unemployed?				
Very concerned	32 (47.8)	19 (33.3)	16 (23.9)	**0.052**
Quite concerned	24 (35.8)	17 (29.8)	30 (44.8)	
**Health conditions**				
What is your health status, from 0 to 100? Mean (SD)	79.9 (19.4)	78.1 (20.4)	78.7 (20.0)	0.880
In the last month, how often have you been able to concentrate?				
Same as usual	45 (67.2)	40 (70.2)	48 (71.6)	0.516
In the last month how often have you felt that you are playing a useful role?				
Same as usual	54 (80.6)	40 (70.2)	46 (68.7)	0.518
Less than usual	10 (14.9)	11 (19.3)	13 (19.4)	
In the last month how often have you lost a lot of sleep because of your worries?				
Something more than usual	29 (43.3)	16 (28.1)	21 (31.3)	0.273
In the last month how often have you felt constantly under pressure?				
No more than usual	33 (49.3)	29 (50.9)	41 (61.2)	**0.033**
No more than usual	37 (55.2)	32 (56.1)	35 (52.2)	0.519
During the last 12 months, have you suffered any injury or damage due to a work-related accident?				
No	62 (92.5)	51 (89.5)	60 (89.6)	0.792

Bold values are statistically significant.

SD = standard deviation.

The work-health relationship was analyzed through first-level coding and analytical interpretations with a phenomenological approach. Verbatim transcriptions exceeding 40,000 words were made, reaching data saturation at participant number 23 (out of 191). The following categories of analysis were defined 1) challenges during the pandemic, 2) work concerns and 3) work well-being during the pandemic.

### CHALLENGES DURING THE PANDEMIC

Narratives in this category focused on the need for a living wage, the view of uncertainty in everyday life, a resilience system, and support networks, as well as innovation initiatives.

*P12 “We [street clothes vendors] had to generate a new way of selling [our products] through WhatsApp: taking pictures and asking family or friends [so] they could see [our products]. [...] Then we had an excuse to leave the house and deliver the orders [clothes]”* (37-year woman, informal worker selling clothes, San Cristóbal, 20 de Julio area).

### WORK CONCERNS

In this category, concepts underlying the following were identified: working without being fined, regaining confidence, and financial gain from street vending. At least 78% of participants reflected on the need to change jobs and the fear of future employment. *P19 “This work is just to survive, because you [as an informal worker] don’t have access to health care, to a pension; to work like this is to be subjected to poverty”* (40-year man, street vendor selling groceries, Kennedy locality, Patio Bonito area).

### WORKPLACE WELL-BEING DURING THE PANDEMIC

This category focused on the transition during the pandemic and the new normality. The analysis was based on infection prevention measures and providing customers with a sense of security regarding the products being sold. *Q2. “In our case [as informal workers], isolating ourselves was the most useful prevention measure. The food [being sold] is no longer served on plates, so we had to buy polystyrene containers and plastic bags”* (45-year woman, street food vendor, Kennedy locality, Patio Bonito area).

## DISCUSSION

The present study was focused on analysis of working and health conditions of a group of street vendors of Bogotá, Colombia, in the context of post-COVID-19 pandemic times, also commonly known as the “new normality”. To achieve this objective, the study population was contextualized in a geopolitical area where street vendors were seriously affected by the social distancing and human mobility restrictions imposed to control the COVID-19 pandemic, and where informal workers are known to be exposed to a higher risk of labor vulnerability and social changes.^17,18^ In addition, in order to perform a more detailed analysis, a mixed methods research was carried out using theoretical points of reference about the work-employment-health relationship, but also comprehensively understanding participants’ context of by semi-structured interviews and qualitative categorical analyses that led to results adjusted to reality.

Our findings regarding participants’ sociodemographic characteristics are similar to those reported by several studies conducted in other countries. For example, a study conducted in Ecuador described an increase of informal employment during the pandemic and in the post-pandemic period, especially among adults older than 24 years, reaching an informal employment rate of 75.9% during the 1st days of the post-pandemic period,^19^ and another study carried out in Bangladesh reported that most of the informal workers included had a low level education and that gender distribution in the sample was similar.^20^

In our study, an association was found between informal workers’ work activity and affiliation to the subsidized social security and health scheme (p = 0.012); moreover, more than 89% of the workers were not affiliated to other social protection systems. Although, as noted above, this finding has already been described in similar studies, its discussion in the context of the COVID-19 pandemic is necessary, as it has a number of individual, community and societal implications related to the risks to which informal workers are exposed. Several of the above ideas have been addressed in the study by Busso et al.,^21^ which evidenced a structural problem characterized by insufficient coverage of social protection systems, a fragmented response by governments to the economic difficulties faced by informal workers, and the implementation of only temporary solutions to existing problems of health coverage, treatment and monitoring.

In relation to the working, employment and health conditions of street vendors, several aspects were identified that need to be worked on in future studies. In the three groups established (food, miscellaneous items, and clothing), most of the informal workers stated that they cannot even take a day off from work (including vacation) deliberately and, even more worryingly, that they cannot get sick or take additional time off due to the economic and social repercussions this entails for them and for their families. Undoubtedly, this situation is contrary to the concept of decent work, goes against the concept of quality of life, and leads to outcomes that impact areas such as economy, health, and social structure.^22^

The impact on participants’ working conditions in the post-pandemic period is related to the perception of perceived income (decrease in their income in the new context of normality) and perception of unfairness in the wage-work performance relationship (p = 0.045). These results confirm the findings described in a study conducted in Bangladesh, where 98% of informal workers experienced a reduction in their income.^20^ Another study, this time in Indonesia, reported that at least 50% of its informal workers believed that their income had decreased by more than half, exposing them to complex unmet needs.^23^ Likewise, the results of an investigation in 11 cities around the world found that economic losses also include a decrease in job opportunities and working hours.^24^

In the results, a contradiction is evident in the dimension of health conditions, since a high score is observed in general health but from adverse conditions such as: a neglect of health in favor of an unfair salary, high workloads and extensive work schedules, sleep difficulties and above all the constant need to satisfy basic needs. This is also identified in the qualitative responses when workers modify their self-care in favor of work activities. In this sense, Delgado-Enciso et al.^25^ and Romero-Michel et al.^26^ reported that many of the street vendors included in their studies would prefer to be infected with the virus rather than stop working, which is related to the importance of having sufficient solvency to cover basic needs in this population and to the fact of not receiving support or economic aid or not being able to find other solutions to cover their basic needs.

Undoubtedly, the qualitative results provided a much broader view of the phenomenon to be studied, and the following challenges were identified: the need to earn at least a living wage, the need to establish support networks with other street vendors, and the creation of innovation networks/job opportunities. Resilience, previously identified in studies related to occupational health in informal workers, is a necessary aspect to discuss and highlight.^27^ In that sense, different studies confirm not only the resilience capacity but also the skills that these workers have in terms of creativity and creation of organized groups to face adversity.^24,27^ For example, Singh & Kaur,^28^ in a study that analyzed the narratives of informal workers in India in the midst of the pandemic, considered a set of additional skills and intersectional aspects that informal workers had to face and overcome such as economic inequalities, poverty and subsistence at home, food insecurity, among others.

The previous discussions lead us to comment on the work of Moussié & Alfers,^29^ with which we agree on the fact that, due to their low income, informal workers cannot afford to pay or receive health care; it is therefore necessary to review the policies and programs directed specifically at populations that are vulnerable or have low social stability. Additionally, they show the importance of taking a position that moves away from the conception of economic regulation and focuses on supporting local economies, since this will have a positive impact on people’s health, well-being, and quality of life conditions.^27,30^

Several studies have reflected on the worsening of the precariousness of informal workers during the COVID-19 pandemic, to which we add the concept of “ultra-precariousness”, defined as the worsening of the employment conditions of these workers evidenced by a higher job insecurity and a higher frequency of adverse working conditions, and the urgent need of going beyond ensuring their enrollment in social protection systems and establishing spaces for discussing development agendas with informal workers, their families and relatives.^20,22,27^

This research has several strengths and limitations. Among its strengths are 1) study designed to understand a phenomenon from both a theoretical basis and phenomenological perspective of reality; and 2) to our knowledge, it is the first study to show a relationship between work-employment-health and quality of life in informal workers in the midst of COVID-19. On the other hand, limitations include: 1) not having a large number of street vendors, this limited by logistical problems and the final scope of the study; 2) the design of the study, since it is a snapshot of the moment and not a follow-up of conditions; and 3) respondent participant bias, since it may influence the fact that some participate and set a pre-determined trend.

## CONCLUSIONS

The working and employment conditions of street vendors include several characteristics that expose them to closer contact with people and to greater workload, not to mention the lack of social protection and their high vulnerability to change. The findings reported here show that these people are in a precarious situation that worsened in the context of the COVID-19 pandemic, a situation that is explained by the reduction in their income and the consequent impossibility of satisfying their basic needs, and the feelings of fear or anxiety about their future.

The reactivation of economic and social activities in Colombia and LAC has not effectively considered informal workers, as shown not only by informal work rates in all countries of LAC, but also by the social perceptions of these workers, which in this study focused on earning at least a living wage, resilience in the face of adversity, the lack of job opportunities, and disregard one’s health to meet daily needs. Instead of focusing on aspects such as the inclusion of informal workers in the banking system or in the formal economy, new approaches to the complexity of this population in LAC must look how to effectively integrate these workers in inclusive social care, social protection, and the health care system that promote social and labor development.
